# Socio-economic status influences the relationship between obesity and antenatal depression: Data from a prospective cohort study

**DOI:** 10.1016/j.jad.2016.05.061

**Published:** 2016-09-15

**Authors:** E. Molyneaux, D. Pasupathy, L.C. Kenny, L.M.E. McCowan, R.A. North, G.A. Dekker, J.J. Walker, P.N. Baker, L. Poston, L.M. Howard, on behalf of the SCOPE consortium

**Affiliations:** a Section of Women's Mental Health, Institute of Psychiatry, Psychology & Neuroscience, King's College London, UK; b Division of Women's Health, Women's Health Academic Centre, King's College London and King's Health Partners, UK; c Irish Centre for Fetal and Neonatal Translational Research (INFANT) and Department of Obstetrics and Gynaecology, University College Cork, Republic of Ireland; d Department of Obstetrics and Gynaecology, University of Auckland, New Zealand; e Department of Obstetrics and Gynaecology, Lyell McEwin Hospital, University of Adelaide, Australia; f Reproduction and Perinatal Health Research Group, University of Leeds, St James University Hospital, Leeds, UK; g College of Medicine, Biological Sciences and Psychology, University of Leicester, UK

**Keywords:** SCOPE cohort, screening for pregnancy endpoints cohort study, SES, socio-economic status, BMI, body mass index, EPDS, Edinburgh postnatal depression scale, Obesity, Antenatal depression, Socio-economic status

## Abstract

**Background:**

Obesity has been associated with increased risk of antenatal depression, but little is known about this relationship. This study tested whether socio-economic status (SES) influences the relationship between obesity and antenatal depression.

**Methods:**

Data were taken from the Screening for Pregnancy Endpoints (SCOPE) cohort. BMI was calculated from measured height and weight at 15±1 weeks' gestation. Underweight women were excluded. SES was indicated by self-reported household income (dichotomised around the median: low SES ≤£45,000; high SES >£45,000). Antenatal depression was defined as scoring ≥13 on the Edinburgh Postnatal Depression Scale at both 15±1 and 20±1 weeks' gestation, to identify persistently elevated symptoms of depression.

**Results:**

Five thousand five hundred and twenty two women were included in these analyses and 5.5% had persistently elevated antenatal depression symptoms. There was a significant interaction between SES and BMI on the risk of antenatal depression (p=0.042). Among high SES women, obese women had approximately double the odds of antenatal depression than normal weight controls (AOR 2.11, 95%CI 1.16–3.83, p=0.014, adjusted for confounders). Among low SES women there was no association between obesity and antenatal depression. The interaction effect was robust to alternative indicators of SES in sensitivity analyses.

**Limitations:**

1) Antenatal depression was assessed with a self-reported screening measure; and 2) potential mediators such as stigma and poor body-image could not be examined.

**Conclusions:**

Obesity was only associated with increased risk of antenatal depression among high SES women in this sample. Healthcare professionals should be aware that antenatal depression is more common among low SES women, regardless of BMI category.

## Highlights

• High SES obese women had twice the odds of antenatal depression than high SES normal weight controls.
• There was no significant association between obesity and antenatal depression among low SES women.
• Antenatal depression was substantially more common among low SES women, regardless of BMI category.

## 1 Introduction

Approximately 20% of women in the UK and USA are obese when they become pregnant (Fisher et al., 2013, Heslehurst et al., 2007). A recent systematic review and meta-analysis showed that obese women are more likely to experience antenatal depression than normal weight women (Molyneaux et al., 2014), but the meta-analysis did not adjust for confounders or examine factors that might influence this relationship. There are limited data in pregnancy but potential mechanisms of the relationship between obesity and depression include inflammation (Miller et al., 2009) and psychosocial factors such as poor body image and stigma (Preiss et al., 2013). Some studies in non-pregnant adults have suggested that the relationship between obesity and depression may be altered by socio-economic status (SES), with obesity and depression more strongly associated among women of higher SES versus lower SES (Moore et al., 1962, Simon et al., 2006). It is not known if there are similar effects of SES during pregnancy. This study used data from the Screening for Pregnancy Endpoints (SCOPE) cohort to examine the effect of SES on the relationship between BMI and antenatal depression.

## 2 Methods

### 2.1 Study population

Data were taken from the SCOPE cohort which recruited healthy nulliparous women with singleton pregnancies from study centres in New Zealand (Auckland), Ireland (Cork), Australia (Adelaide) and the UK (London, Leeds and Manchester) between November 2004 and January 2011. Women were recruited at 14–16 weeks' gestation and followed up to delivery. Women were excluded from the cohort if they were at particularly high risk of pre-eclampsia, small for gestational age delivery or spontaneous preterm birth due to underlying medical conditions or gynaecological history, or had received interventions that might have modified pregnancy outcome (for full details, see McCowan et al., 2007). In addition, underweight women (1.5% of the sample) and women with fetal loss before 22 weeks’ gestation were excluded from the sample for these analyses.

## 3 Measures

### 3.1 Antenatal depression

Antenatal depression was assessed with the Edinburgh Postnatal Depression Scale (EPDS; Cox et al., 1987) at 15±1 and 20±1 weeks' gestation using a validated cut-off of ≥13 (Murray et al., 1990). In this study, antenatal depression was defined as scoring ≥13 on the EPDS at both 15±1 and 20±1 weeks’ gestation; the use of repeated measurements identifies women with persisting symptoms of depression and has a higher positive predictive value for depression (based on diagnostic interview) than a single EPDS assessment (Nyklíček et al., 2004). EPDS score was missing for 24 women (0.4% of the sample) at 15±1 weeks' gestation and 149 women (2.7%) at 20±1 weeks' gestation. Missing observations at 20±1 weeks' gestation were imputed with the participant's EPDS score from 15±1 weeks’ gestation if available, otherwise missing EPDS observations were imputed with the median score.

### 3.2 Body mass index (BMI)

BMI was calculated from measured height and weight at 15±1 weeks’ gestation and categorised as normal weight (18.5–25 kg/m^2^), overweight (25–30 kg/m^2^) or obese (≥30 kg/m^2^). There was no missing data for BMI.

### 3.3 Socio-economic status (SES)

For the main analyses, SES was based on self-reported pre-tax household income, converted between currencies. This was dichotomised around the median income boundary (low SES: ≤£45,000, high SES: >£45,000; equivalent to $74,000 AUD/NZD or €63,000). Missing income data (n=551, 9.8%) was imputed using expectation maximisation based on employment status, ethnicity, education, occupation, and socio-economic index. Sensitivity analyses were performed using alternative indicators of SES (see Statistical methods).

### 3.4 Confounders

Potential confounders were self-reported at 15±1 weeks' gestation: age, ethnicity, marital status, education, socio-economic index (based on current or previous occupation; Galbraith et al., 1996), employment status, pre-pregnancy smoking, pre-pregnancy alcohol consumption and previous pregnancy loss. The measurement of these variables is described in Supplementary data 1.

### 3.5 Statistical methods

Statistical analyses were conducted using Stata 12. Logistic regression was used to calculate the odds of antenatal depression for overweight and obese women compared with normal weight controls, adjusting for confounders. Centre of recruitment was also included as an *a priori* confounder in all adjusted analyses. The interaction of BMI category and SES on the risk of antenatal depression was tested. If the interaction effect was statistically significant based on the Wald test, the associations between BMI category and antenatal depression were re-calculated separately for high and low SES women. The association between each unit increase in BMI and risk of antenatal depression was also calculated. Sensitivity analyses were performed to examine the robustness of the interaction effect to different indicators of SES: 1) using a lower household income cut-off (low SES: ≤£30,000, high SES: >£30,000); 2) using SES based on occupation (low SES: manual, service or sales jobs, high SES: technical, professional or managerial jobs), and 3) using continuous BMI multiplied by income in increments of £15,000 to form the interaction term.

## 4 Results

Eight thousand five hundred and thirty one women were approached for the SCOPE cohort and 5628 eligible women participated in the baseline interview (flow diagram given in Supplementary data 2). Participants were excluded from these analyses if they were underweight (n=84) or experienced fetal loss before 22 weeks’ gestation (n=22), leaving 5,522 women included in this study. Characteristics of the sample are shown in Table 1. The majority of women (64.7%) were 25–34 years old. Most participants (90.1%) were of white ethnicity and were married (58.9%) or cohabiting (31.7%). Under half had graduated from university (43.4%) and 85.6% were in paid work. 10.1% reported household income under £15,000/year (or equivalent), whilst 13.9% reported household income over £75,000/year. Just over half of the sample were normal weight (56.4%; n=3,113), 28.5% (n=1,571) were overweight and 15.2% (n=838) were obese.

**Fig. 1:**
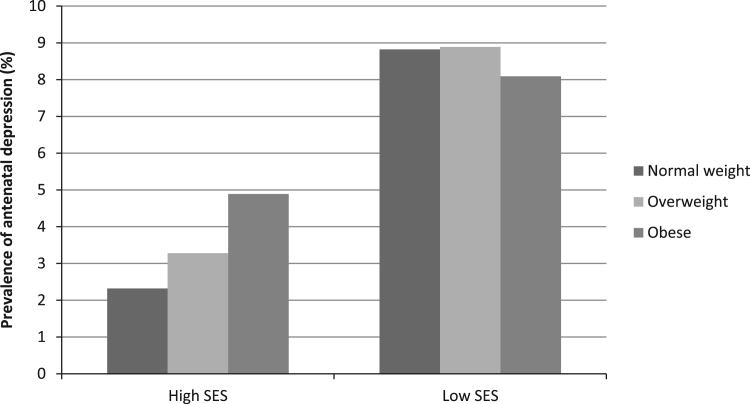
Prevalence of antenatal depression by BMI category for high SES women and for low SES women.

At 15±1 weeks' gestation, 11.8% (n=654) of participants had EPDS scores ≥13 and at 20±1 weeks’ gestation, 9.3% (n=513) had EPDS scores ≥13. In total, 5.5% (n=303) of participants had EPDS scores ≥13 at both time points and were classified as having antenatal depression for these analyses. The prevalence of antenatal depression was 5.0% among normal weight women, 5.7% among overweight women and 6.7% among obese women. Unadjusted logistic regression showed no significant associations between overweight or obesity and antenatal depression, compared with normal weight controls (overweight OR 1.14, 95%CI 0.88–1.49, p=0.322; obesity OR 1.35, 95%CI 0.98–1.85, p=0.063). There was evidence for a significant interaction of BMI and SES on antenatal depression (Wald test p=0.042) so the sample was divided into high SES and low SES women for further analyses. The prevalence of antenatal depression increased with increasing BMI for the high SES women (normal weight 2.3%, overweight 3.3%, obese 4.9%) but not for the low SES women (normal weight 8.8%, overweight 8.9%, obese 8.1%), among whom antenatal depression was more common (see Fig. 1).

Among high SES women, obesity was significantly associated with higher odds of antenatal depression (OR 2.16, 95%CI 1.23–3.80, p=0.007; AOR 2.11, 95%CI 1.16–3.83, p=0.014), compared with high SES normal weight controls. There was no significant association between overweight and antenatal depression among high SES women (OR 1.43, 95%CI 0.88–2.30, p=0.148; AOR 1.45, 95%CI 0.88–2.37, p=0.144). Among low SES women, there were no associations between obesity and antenatal depression (OR 0.91, 95%CI 0.62–1.33, p=0.627; AOR 0.86, 95%CI 0.57–1.30, p=0.468) or overweight and antenatal depression (OR 1.01, 95%CI 0.73–1.40, p=0.956; AOR 0.99, 95%CI 0.71–1.40, p=0.968), both compared with low SES normal weight controls. In addition, each unit increase in BMI was associated with significantly increased risk of antenatal depression among high SES women (AOR 1.06, 1.02–1.11, p=0.004) but not among low SES women (AOR 0.99, 0.96–1.02, p=0.434).

Three sensitivity analyses were conducted with alternative indicators of SES, two using different categorisations of household income and one based on occupation. Each analysis showed a significant interaction between BMI and SES on the risk of antenatal depression (Wald tests for interaction effects: p=0.002 to p=0.009).

## 5 Discussion

There was a significant interaction between BMI and SES on the risk of antenatal depression in this sample. Among high SES women (household income >£45,000 per year), the odds of antenatal depression were approximately twice as high for obese women than for normal weight controls, even after adjustment for confounders. In contrast, there was no association between obesity and antenatal depression among low SES women. However, the prevalence of antenatal depression was substantially higher among low SES women than high SES women, regardless of BMI category.

The influence of SES on the relationship between BMI and depression has been reported for non-pregnant women (Moore et al., 1962, Simon et al., 2006, Stunkard et al., 2003) but, to our knowledge, had not previously been observed among pregnant women. Obesity did not appear to be a risk factor for antenatal depression among low SES women in this sample, potentially due to the dominance of other risk factors for depression in this group, such as childhood deprivation and stressful life events. In addition, lower SES women, among whom obesity is more common, may experience less obesity-related stigma than high SES women. Overweight and obese high SES women have been found to report greater body image dissatisfaction than lower SES women of the same BMI (McLaren and Kuh, 2004, Wardle and Griffith, 2001), and poor body image may mediate the effect of obesity on depression (Gavin et al., 2010). Obesity-related stigma and body image were not assessed in this cohort but should be examined in future research. The findings of this study also need to be replicated. In addition, obesity in pregnancy has been associated with adverse effects on child development (including increased risk of behavioural disorders; Sullivan et al., 2015) and future research should examine the influence of comorbid antenatal depression on these outcomes.

This study had a number of strengths including the prospective study design, high retention rate in the SCOPE cohort, and the use of objectively measured height and weight to calculate BMI. Symptoms of antenatal depression were self-reported using the EPDS which is a validated screening tool for depression in pregnancy but is not a diagnostic assessment, limiting the conclusions which can be drawn (Kozinszky et al., 2015). However, self-reported measures are more feasible for large samples and less burdensome for participants. In addition, the identification of persistently elevated symptoms of depression (EPDS ≥13 at both 15±1 and 20±1 weeks' gestation) was a strength of this study and has been shown to have a higher positive predictive value for major depressive disorder than a single EPDS assessment (Nyklíček et al., 2004). A number of biomarkers for antenatal depression have also been examined (Serati et al., 2016), but it is unclear how these are influenced by obesity.

Limitations of this study include limits to generalisability based on the exclusion of parous women and those with certain chronic medical conditions from the SCOPE cohort. In addition, 90% of participants were of white ethnicity and over half of participants reported household incomes greater than £45,000. This was a limitation for the main analyses as income was dichotomised around the median, meaning that the ‘low’ SES group included women with household income up to £45,000 (or equivalent). However, the interaction of SES and BMI on the risk of antenatal depression was also observed in three sensitivity analyses using a lower household income boundary (£30,000), using income in increments of £15,000, and using SES based on occupation. The interaction effect therefore appears robust to different indicators of SES. Finally, women with antenatal depression have often experienced previous episodes of depression (Patton et al., 2015) which may have preceded the development of obesity. It is therefore not possible to draw conclusions about the direction of causality for the relationship between obesity and antenatal depression among high SES women in this sample.

## 6 Conclusions

In this study, women with lower SES had substantially higher prevalence of antenatal depression than high SES women, regardless of BMI category. This is in keeping with the well-established association between socio-economic deprivation and depression. There was no association between obesity and antenatal depression among low SES women in this sample, but among high SES women the odds of antenatal depression were approximately twice as high for obese women compared with normal weight controls. These findings add to the broader literature from non-pregnant adults suggesting that SES may influence the relationship between BMI and depression.

## Contributors

Authors EM, DP, LP and LMH designed the study. EM and DP conducted the statistical analyses and EM DP, LP and LMH drafted the manuscript. All authors contributed to and have approved the final manuscript.

## Figures and Tables

**Table 1:** Sample characteristics.

		**n (%)**
**Age (years)**	<20	381 (6.9)
	20–24	858 (15.5)
	25–34	3575 (64.7)
	35–39	620 (11.2)
	≥40	88 (1.6)

**Ethnicity**	White	4975 (90.1)
Asian/Indian	292 (5.3)

	Maori/Pacific Islander	113 (2.1)

Other	142 (2.6)

**Marital status**	Married	3251 (58.9)
Cohabiting	1748 (31.7)
Single or separated	523 (9.5)
**Educational level**	No	3125 (56.6)
(graduated from university)	Yes	297 (43.4)

**Household income**	<£15k	557 (10.1)
£15–45k	1903 (34.5)
£45–75k	2296 (41.6)
>£75	766 (13.9)
**Socioeconomic index; median (IQR)**		45 (28–50)

**Occupational status**	Paid work	4729 (85.6)
Student	173 (3.1)
Homemaker	158 (2.9)
Not in paid work	462 (8.4)

**County of recruitment**	Australia	1122 (20.3)
Ireland	1749 (31.7)
New Zealand	2001 (36.2)
UK	650 (11.8)
