# Supplementary material for: Socio-economic status influences the relationship between obesity and antenatal depression: Data from a prospective cohort study
Source: J Affect Disord. 2016 Sep 15;202:124–7. doi: 10.1016/j.jad.2016.05.061 (PMC4957541; doi:10.1016/j.jad.2016.05.061)

Supplementary Data 1: Assessment and coding of confounding variables

Variable	Assessment and coding of each variable and details of missing data
**Age**	Participants reported their age at recruitment into the study. The association of age with antenatal depression was non-ordinal so age was coded in categories for the analyses (<20 years, 20-25, 25-30, 30-35, 35-40, ≥40). No participants had missing data for this variable.
**Ethnicity**	Participants reported their main ethnicity which was categorised as White, Asian/Indian, Maori/Pacific Islander, African or Other. No participants had missing data for this variable.
**Marital status**	Marital status was self-reported at the baseline study visit as single, married, defacto married, separated, divorced or same sex partner. Owing to small number of women in some groups, this was categorised as married, cohabiting (including defacto married and same sex partner) and single (including single, separated or divorced). No participants had missing data for this variable.
**Highest educational level**	Highest educational level was categorised as a dichotomous variable indicating whether or not each woman had graduated from university. No participants had missing data for this variable.
**Socio-economic index**	Women self-reported their current or previous occupation. This was used to define their socio-economic index based on the New Zealand Socioeconomic Index guide (Galbraith, Jenkin et al. 2003) which gives each reported occupation a score (ranging from 18 for labourers to 90 for senior business people). This data also enables classification based on occupation which was used for a sensitivity analysis to examine the interaction of BMI and SES (see Statistical Methods). There was no missing data for this variable.
**Job status**	Women reported their current job situation which was classified as paid work (including women reporting full or part time jobs), student, home-maker and not in paid work (including women who were unemployed, unable to work due to sickness or doing voluntary work). There was no missing data for this variable.
**Smoking status**	Women reported the average number of cigarettes they smoked per day in the three months prior to pregnancy. No participants had missing data for this variable.
**Alcohol consumption**	Women reported the average number of units of alcohol they consumed per week in the three months prior to pregnancy. No participants had missing data for this variable.
**Previous pregnancy loss**	Women self-reported the number of previous miscarriages they had had. There was no missing data for this variable.

Supplementary Data 2: Cohort flow diagram

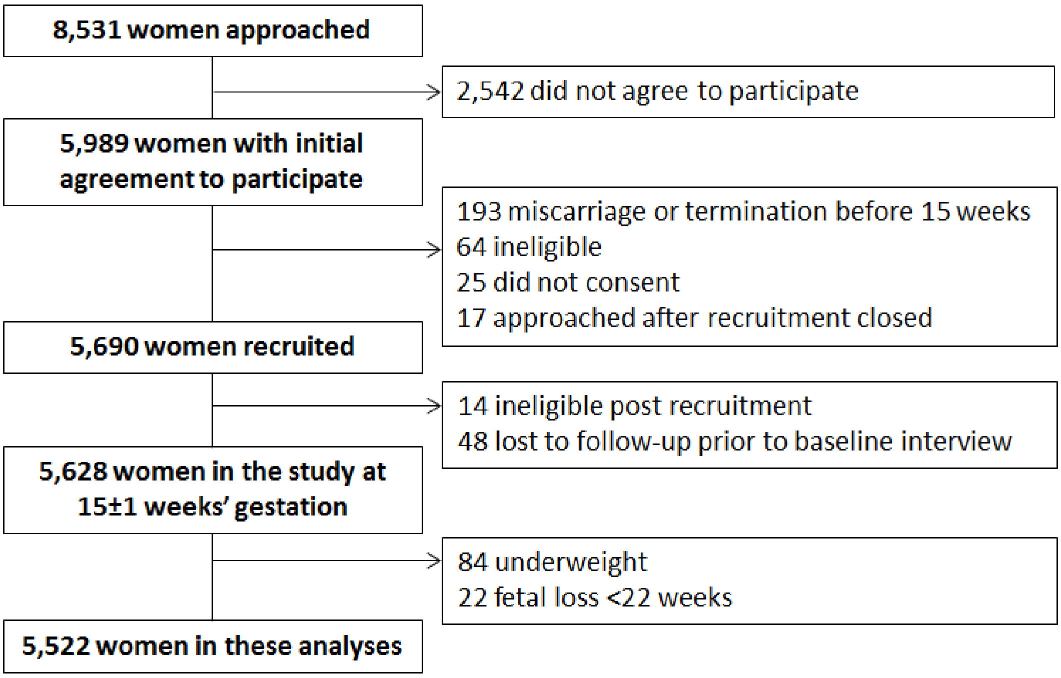

Supplement: Supplementary material [file mmc1.pdf]
